# THz Spectroscopic Investigation of Wheat-Quality by Using Multi-Source Data Fusion

**DOI:** 10.3390/s18113945

**Published:** 2018-11-14

**Authors:** Hongyi Ge, Yuying Jiang, Yuan Zhang

**Affiliations:** 1 Key Laboratory of Grain Information Processing & Control, Ministry of Education, Henan University of Technology, Zhengzhou 450001, China; gehongyi@haut.edu.cn (H.G.); jiangyuying11@163.com (Y.J.); 2 College of Information Science and Engineering, Henan University of Technology, Zhengzhou 450001, China; 3 Key Laboratory of Henan Province for Grain Photoelectric Detection and Control, Zhengzhou 450001, China; 4 National Engineering Laboratory for Wheat & Corn Further Processing, Zhengzhou 450001, China

**Keywords:** THz spectroscopy, Multi-Source Data Fusion, support vector machine, DS evidence theory, wheat quality

## Abstract

In order to improve the detection accuracy for the quality of wheat, a recognition method for wheat quality using the terahertz (THz) spectrum and multi-source information fusion technology is proposed. Through a combination of the absorption and the refractive index spectra of samples of normal, germinated, moldy, and worm-eaten wheat, support vector machine (SVM) and Dempster-Shafer (DS) evidence theory with different kernel functions were used to establish a classification fusion model for the multiple optical indexes of wheat. The results showed that the recognition rate of the fusion model for wheat samples can be as high as 96%. Furthermore, this approach was compared to the regression model based on single-spectrum analysis. The results indicate that the average recognition rates of fusion models for wheat can reach 90%, and the recognition rate of the SVM radial basis function (SVM-RBF) fusion model can reach 97.5%. The preliminary results indicated that THz-TDS combined with DS evidence theory analysis was suitable for the determination of the wheat quality with better detection accuracy.

## 1. Introduction

Wheat is one of the major food sources in the world. It contains starch, protein, carbohydrates, lipids, and is a healthy food source of multiple nutrients and dietary fiber, particularly in many countries where wheat products are primary foods [1,2,3,4]. Inadequate control of the storage conditions can easily lead to mildew, moisture regain, germination, and insect infestation, which all reduce wheat quality [5,6]. Therefore, it is highly desirable to conduct research to obtain a fast and non-destructive detection method for wheat quality. Such a method could also effectively reduce post-harvest losses of grains and improves food safety.

Methods for inspecting quality of stored wheat as reported by domestic and foreign research include [7,8,9,10] chemical methods (measurement of major nutrient content, such as protein, carbohydrates, etc.), electronic nose, machine vision, spectrum detection (infrared spectroscopy). These methods are time-consuming and laborious, with a high consumption of both samples and reagents, and they have limited inspecting capabilities. THz radiation is sensitive to the vibrational states of the entire molecule, which means that terahertz radiation can be used to identify many materials. Due to its unique advantages such as the low energy of single photons and the “fingerprint character”, THz time-domain spectroscopy (THz-TDS) is an effective non-contact measurement technique to analyze the internal composition of a substance. THz-TDS can also provide a direct measurement of the time-resolved amplitude and phase information, which are related to the absorptive and refractive properties of the sample, THz-TDS is currently the most used THz technology because it has important applications in the fields of biomedicine, material science, national defense, and quality control [11,12,13,14,15].

With regards to the qualitative and quantitative analysis of material using THz waves, many researchers have been focusing on the optical and spectral characteristics of the material within the THz band, and they carried out the measurement of the THz absorption coefficient and the refractive index coefficient, as well as an analysis of this single optical parameter. Many studies [16,17,18,19,20,21,22] have reported the combination of THz spectroscopy and chemometric methods (principal component analysis, partial least squares, support vector machines) to achieve a quantitative analysis of materials, and they achieved good results. The research group [23] used THz-TDS technology and the chemometric method to identify samples of different quality. The single-spectrum of wheat grain sample was used as the input to the constructed models, the comparison results demonstrated that the PCA-SVM approach can obtain satisfactory prediction accuracy for identifying the four types of wheat grain. However, the recognition rates for normal and germinated wheat were relatively high, while the recognition rates for moldy and worm-eaten wheat were low. The recognition rates for different models vary considerably.

This paper aimed to inspect different wheat qualities through the combination of multi-source information fusion technology and the THz-spectrum identification method in order to improve the recognition accuracy for wheat-quality classification. The wheat quality defects non-destructive detecting model used multi-source information fusion technology by fusing information of the absorption and refractive index spectrum of wheat samples of different quality. In the decision-making layer of fusion, the optical parameters were trained using an RBF kernel-function SVM, and the sub-classifier for wheat of varying quality was generated. The output results of the sub-classifier were fused using DS evidence theory. In the feature layer of fusion, the SVM method was used to construct a wheat-quality classification model using a feature layer, and the validation was carried out. Finally, the optimal fusion and classification methods of multi-source information for wheat quality were determined. These can be applied to identify and detect storage induced quality defects, and to provide the foundation for the application of THz technology for grain quality detection.

## 2. Experimental Methods

### 2.1. Experimental Setup

The spectra of the samples were measured using a Zomega-Z3 type THz-TDS system, which is located in the key laboratory grain information processing and control, Ministry of Education, Henan University of Technology (Zhengzhou, China). The detailed description of the device and measurement system are described in the literature [20,23]. A femtosecond fiber laser was used as light source, with a center wavelength of 780 nm, a repetition rate of 80 MHz, and a pulse width of 100 fs. The related system parameters of the Z3-type THz-TDS are shown in Table 1, and the acquisition mode for the sample’s spectral data was the transmission mode.

All spectroscopic measurements of the wheat samples were performed at room temperature, 292 K. During the entire measurement, in order to reduce the effect of air moisture on the absorption of THz wave, the THz light path was filled with dry nitrogen. In addition, in order to reduce the measurement error, each sample was measured 5 times and the mean value was calculated.

### 2.2. Sample Preparation and Parameter Extraction

The preparation process of the wheat samples with different qualities was reported in the literature [23]. For sprouting wheat, the wheat samples were cleaned and soaked in water for 2 h and drained, and then enclosed in containers, and incubated at 25 °C for 18 h; For moldy wheat, the wheat samples were moistened at a humidity of 28% and were put in a circular Petri dish, and then put into an incubator box at a constant temperature of 20 °C for 2 days; For worm-eaten wheat, the nutrient component was partially eaten by corn elephants. The culture samples were used for measurement. The moisture content of the samples was about 12.5%, measured by the grain moister sensor. The main component of wheat is measured, the protein content is 12.31% (moldy wheat), 10.22% (worm-eaten wheat), 14.97% (germinated wheat), and 15.43% (normal wheat) respectively, and the fat content is 5.01% (moldy wheat), 3.15% (worm-eaten wheat), 5.27% (germinated wheat), and 6.76% (normal wheat) respectively. The average protein content is 13.23%, the average fat content is 5.05%, and the standard deviations of protein content and fat content are 0.024 and 0.015. Wheat samples were ground into fine powder, which was subsequently sieved by filtering laws using 200-eye sieves; the wholemeal flour was used and then pressed into 1 mm thick pellets with a pressure of 10 tons for 5 min, all the samples were labeled according to their properties as being worm-eaten, moldy, sprouting, or normal. When the sample was tested with the THz-TDS system, the THz pulse was absorbed and scattered by the sample. After a fast Fourier transform (FFT), the time domain signal of the sample was transformed into the frequency domain signal [24,25].

(1) $$\tilde{E}(\omega )=A(\omega )e^{-i\phi (\omega )}=\int dtE(t)e^{-i\omega t}$$

Here, A(ω) is the amplitude ratio of the sample to reference signal, ϕ(ω) is the phase difference between sample and reference signal, $\phi (\omega )=\phi_{s}(\omega )-\phi_{ref}(\omega )$. The absorption coefficient of sample (α) and refractive index coefficient (n) can be calculated by comparing the sample spectrum with the reference spectrum.

(2) $$\alpha =\frac{1}{d}\ln\frac{A_{r}}{A_{s}}$$

(3) $$n=1+\frac{(\phi_{s}(\omega )-\phi_{r}(\omega ))C}{\omega d}$$

where, $A_{r}$ and $A_{s}$ are the amplitudes of the reference signal and the sample signal, respectively. $\phi_{s}(\omega )$ and $\phi_{r}(\omega )$ are the phases of the sample signal and the reference signal, respectively. The variable ω is the frequency, C is the speed of light, and d is the thickness of the sample.

### 2.3. The Multi-Source Information Fusion Method

Multi-source information fusion is useful to analyze, control, and comprehensively process the multiple information resources for different times and space parameters, as well as to obtain new and more useful information to recognize and classify the measured object [26,27,28]. Multi-source information fusion can be divided into the following: data layer fusion, decision layer fusion, and feature layer fusion [29,30]. In this paper, the feature layer and the decision layer were used to fuse the spectral data.

**Figure 1:**
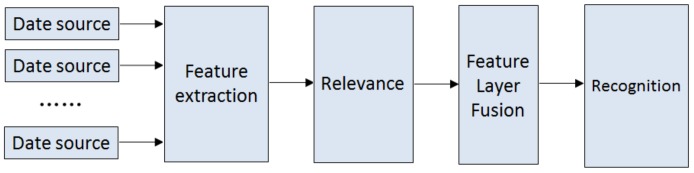
Feature layer information fusion.

Feature layer fusion extracts the feature vector from the collected original data (absorption coefficients and refractive index coefficients of the samples). It not only performs the compression of data and reduces the amount of interference data, but also maintains the important information contained in the original data. The pattern recognition method is then used for comprehensive analysis and treatment, which enables high recognition accuracy. The fusion process was shown in Figure 1.

SVM is a learning method based on statistical learning theory, which combines the advantages of non-linearity, high-dimensionality, and a good generalization performance. It not only improves the accuracy of the fusion results, but also improves the utilization efficiency of input model data greatly. This has become a popular topic of current research. The specific process of SVM is described in detail in the literature [23,31,32].

Decision layer fusion serves to fuse the recognition results of multiple classification models for measurement targets, and it then executes the final reasoning and decision-making process [29]. Because the decision layer fusion performs the compression of the original data information, it is very flexible, requires a small amount of communication data, has a strong fault tolerance, and has a lower transmission bandwidth. In addition, it also has a relatively low requirement for the overall system. In this paper, Dempster-Shafer (DS) evidence theory method was used to create the decision-layer fusion model.

**Figure 2:**
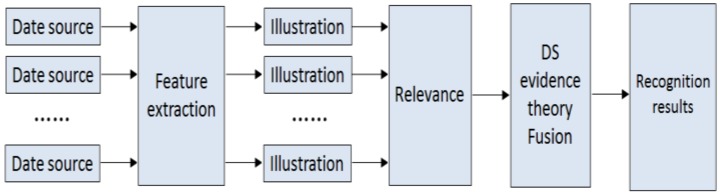
Decision-layer fusion.

DS evidence theory [33,34,35] was first proposed by Dempster in 1967, and then expanded by Shafer and later further developed into DS evidence theory (Dempster/Shafer evidence theory). This method involves imprecise reasoning theory. It belongs to the field of artificial intelligence and has the ability to deal with uncertain information. When dealing with the problem of uncertainty including agnosticism, fuzziness, and randomness, the method with DS evidence combination at its core becomes very effective. DS evidence theory does not need to consider the sequence of multiple pieces of evidence, and can carry out appropriate adjustments according to the object properties during the fusion process. When the evidences are in conflict or in strong correlation, they can be grouped and respectively fused to reduce their impact. The fusion process is shown in Figure 2. The feature information of the sample was obtained, and then the support vector machine [36,37] using the radial basis kernel function was used to train the feature information. Then the corresponding sub-classifiers, RBF-SVM (absorption coefficient) and RBF-SVM (refractive index), were constructed. Finally, DS was used to fuse the output results of the sub-classifiers to obtain the final fusion results.

## 3. Results and Discussion

### 3.1. THz Spectrum Analysis of Wheat of Varying Quality

**Figure 3:**
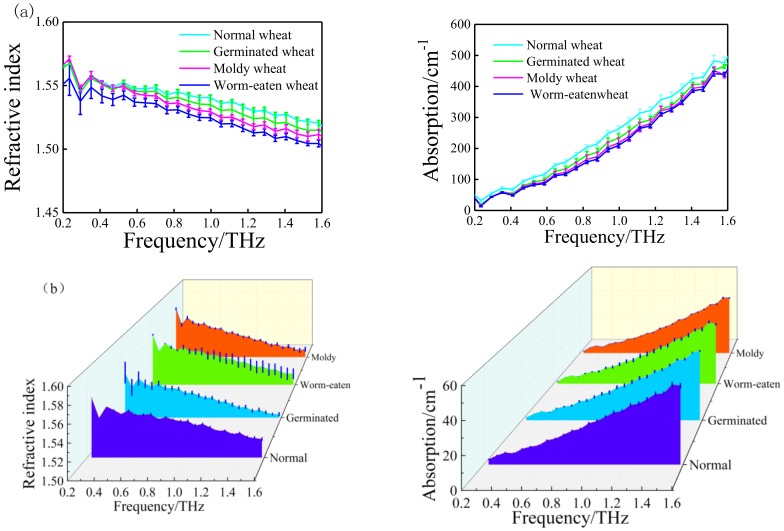
Refractive index spectra and absorption spectra (**a**) of different wheat samples and waterfall plot (**b**).

Due to internal composition changes of moldy wheat, worm-eaten wheat, germinated wheat, and normal wheat, the interaction between molecules changes, and the vibration modes are different. In this case, the response characteristics are in the range of 0.2–1.6 THz. Four types of wheat samples were selected in this experiment, and 60 samples for each type were randomly selected for tests. The THz spectra of 240 wheat samples were measured using the THz-TDS. Each wheat sample was scanned 5 times, and the sample spectrum was the average of 5 scanning spectra in the range of 0.2–1.6 THz. The refractive index and absorption coefficient of the wheat samples are calculated from the THz spectra in the transmission mode, the validity and accuracy of all the measured spectra was evaluated by the standard deviations. Error bars that contain the mean and standard deviations for the 60 samples (one type) are shown in Figure 3. The mean values of the absorption spectra of moldy wheat, worm-eaten wheat, germinated wheat, and normal wheat are 2096.767, 2146.663, 2241.059, and 2466.321. The standard deviation ranges varies between 5.6344 and 72.5934. The error bars of absorption spectra at 1.5 THz with 71.2743 standard deviation and refractive index at 0.3 THz with 0.19 standard deviation are more separated from others.

It can be seen from Figure 3 that the refractive index for the different wheat was 1.50–1.56, and the difference is very clear. The refractive index of normal wheat was the highest, and the order of intensity was n_worm-eaten_ wheat < n_moldy_ wheat < n_germinated_ wheat < n_normal_ wheat. The normal wheat sample had the most intense absorption and the highest absorption coefficient for THz waves, which is consistent with the analysis results in the literature [23]. The absorption curve of the sample did not have the obvious characteristic peak, and the differences in the absorption spectra for the wheat samples are not significant; chemometric methods were employed to investigate the relationship between the minor spectral differences and the measured wheat quality defects. In order to improve the detection accuracy for the quality of wheat, multi-source information fusion technology is proposed for the identification of wheat quality defects.

### 3.2. The Wheat Classification Model Based on Feature Layer Fusion

Principal component analysis (PCA) was used to extract the absorption spectrum and refractive index spectrum characteristics of wheat samples with varying quality. SVM was used as a model identification method, and different kernel functions were used to constructed different types of classification models. The main kernel functions were the linear kernel function, the polynomial kernel function, and the radial basis kernel function; they respectively were Linear-SVM, Poly-SVM, and RBF-SVM. The established models were compared to find the best feature layer fusion model.

(1) The establishment of the RBF-SVM fusion model

The calculated spectral information of 240 wheat samples was divided into two groups randomly according to the ratio of 2:1. Among them, the numbers of spectra in the training set and those in the test set were 160 and 80, respectively. The first eight principal component characteristics were fused into the absorption spectrum feature set of the wheat samples, and the first 10 principal components characteristics were fused into the refractive index spectra feature set of the wheat samples. The wheat classification and fusion model based on SVM was created. The RBF kernel function was chosen as the kernel function of the fusion model, and the optimal parameter () of the kernel function was calculated using the grid search optimization algorithm. Table 2 showed the recognition rate and the false judgment number for all types of wheat samples. After model optimization, the recognition rate of the training set of wheat samples with different quality reached 100%, and the recognition rate for the test set was 97.5%.

According to the table, the recognition rates of the established RBF-SVM fusion model for the normal, germinated, moldy, and worm-eaten wheat samples were 100%, 100%, 95.24%, and 96.65%, respectively.

(2) The linear/poly-SVM fusion model

Again, the first eight principal component characteristics were combined into the absorption spectrum feature set of the wheat samples. Then the first 10 principal component characteristics were combined into the refractive index spectra feature set of the wheat samples, and the different quality wheat classification and fusion model based on SVM was obtained. Linear and Poly kernel functions were chosen as the kernel function for the fusion model. Table 3 and Table 4 showed the recognition rate and the false judgment number for all types of wheat samples. After model optimization, the recognition rate for the training set of wheat samples with different quality reached 100%, and the recognition rates for the test set were 93.75% and 90%, respectively.

According to the table, the recognition rates for the established linear/poly-SVM fusion models for normal wheat samples reached 100%. There was a difference between the recognition rates of germinated, moldy, and worm-eaten wheat samples. Furthermore, in the process of testing, there were two false judgments that falsely identified the moldy and worm-eaten wheat as normal wheat. The results showed that the recognition rate of the RBF-SVM fusion model for the four wheat samples is higher than for other models.

### 3.3. The Decision Layer Fusion Model for Wheat Recognition Using DS Evidence Theory

The four wheat samples of normal wheat, germinated wheat, moldy wheat, and worm-eaten wheat were selected as study objects. Based on DS evidence theory, the classification and fusion model for wheat of different quality was established. The establishing process of the model was as follows:

(1) Respectively established the classification probability output of wheat corresponding to the absorption spectra and refractive index spectra for wheat of different quality.

For the absorption spectrum of different qualities of wheat, the first 10 principal components characteristics were combined into an absorption spectrum feature set, and the wheat classification model was established using the SVM pattern recognition method, with the optimal parameters of RBF kernel function, γ and C, being 3.5 and 1.6. Then the probability outputs of the training set and the test set were obtained.

For the refractive index spectrum of different qualities of wheat, the first eight principal components characteristics were combined into spectrum feature set, and the wheat classification model was established using the SVM pattern recognition method with the optimal parameters of RBF kernel function, γ and C, being 3 and 0.92. Then the probability outputs of the training set and the test set for different qualities wheat were calculated.

(2) Established DS evidence fusion rules

The recognition framework of DS fusion model was assumed as $\Theta =\{\alpha_{1},\alpha_{2},\alpha_{3},\alpha_{4},U\}$. Among them, $\alpha_{1},\alpha_{2},\alpha_{3},\alpha_{4}$ represents normal wheat, germinated wheat, moldy wheat, and worm-eaten wheat, U is the wheat of uncertain quality, which may be any of the four types. Belabsorption and Belrefraction, respectively, are the belief functions of SVM classification model for the sample set with the same Θ. Mabsorption and mrefraction are the basic probability assignment functions of the wheat sample absorption spectra and refractive index spectra SVM classifier for the training set of the four types of wheat samples of different quality. Mabsorption and Mrefraction are the basic probability assignments (BPA) of the absorption spectra and refractive index spectra SVM classifier for the test set of four different qualities. $M_{c}$ is the fusion probability function of the wheat samples absorption spectra and refractive index spectra SVM classifier using the fusion of DS evidence theory. The fusion rules are as follows:

(4) $$M_{\mathrm{absorption}}=m_{\mathrm{absorption}}(A)Bel(A)$$

(5) $$M_{\mathrm{refraction}}=m_{\mathrm{refraction}}(A)Bel(A)$$

(6) $$K=\sum_{\begin{array}{l} i=\mathrm{absorption},j=\mathrm{refraction}\\ A_{i}\cap t_{j}=\phi \end{array}}M_{\mathrm{absorption}}(A_{i})M_{\mathrm{refraction}}(A_{j})$$

(7) $$M_{c}(A)=\sum_{\begin{array}{l} i=\mathrm{absorption},j=\mathrm{refraction}\\ A_{i}\cap t_{j}=A \end{array}}M_{\mathrm{absorption}}(A_{i})M_{\mathrm{refraction}}(A_{j})/(1-K)$$

Here, A∈Θ. Decision rules: Set $\exists A_{1},A_{2}\in \Theta$, and satisfy:

(8) $$M_{c}(A_{1})=\max\left\{M_{c}(A_{i}),A_{i}\in \Theta \right\},M_{c}(A_{2})=\max\left\{M_{c}(A_{i}),A_{i}\in \Theta \cap A_{i}\neq A_{1}\right\}$$

If it satisfies the conditions $\left\{ \begin{array}{l} M_{c}(A_{1})-M_{c}(A_{2})>\varepsilon_{1}\\ M_{c}(\Theta )<\varepsilon_{2}\\ M_{c}(A_{1})>M_{c}(\Theta ) \end{array}\right.$, among which $\varepsilon_{1}$ and $\varepsilon_{2}$ are the threshold of judgment, $A_{1}$ can be determined as the final decision.

(3) Recognition results of the classification fusion model for wheat using DS evidence theory

Following the above theory, normal, germinated, moldy, and worm-eaten wheat were selected as study objects in this paper, and through a combination of the absorption and refractive index spectra of the wheat samples, the classification fusion model for wheat quality defects was established using DS evidence theory. The recognition rate of the four wheat samples reached 100% in the training set, and the recognition rate of the test set was relatively low, 96.25%. The recognition rate and the number of false judgment of different wheat samples are shown in Table 5.

According to the table, the recognition rate of the DS evidence theory fusion model for all types of wheat samples in the training set was high, up to 100%. For the test set, the recognition rates for normal wheat and germinated wheat were 100%, while the recognition rates of the moldy and the worm-eaten wheat were relatively low, only 95.24% and 91.3%, respectively. The experimental results indicate that, compared with the recognition rate obtained from the models based only on the absorption spectra or the refractive index spectra, the recognition rate using the DS evidence theory fusion model for wheat of different quality had improved. This is true especially for moldy and worm-eaten wheat samples, and the sample recognition rate has improved significantly.

### 3.4. Comparison of the Different Fusion Models

For the absorption and refractive index spectra of the four types of wheat, normal wheat, germinated wheat, moldy wheat, and worm-eaten wheat, the classification detection fusion model of multiple-spectral indices of wheat was established using DS evidence theory and SVM model with different kernel functions. The results show that the recognition rates of the fusion models are similar, and the prediction accuracies of the fusion models are higher than with the models established using the simple spectrum. In addition, the fusion models were able to accurately recognize the normal wheat samples. The main difference appeared in the germinated, moldy, and worm-eaten wheat samples, where the recognition rate for the fusion model established on RBF-SVM was slightly higher than that using DS evidence theory fusion.

For the PCA-SVM model established with the absorption spectrum only, the recognition rates for normal, germinated, moldy, worm-eaten wheat, and the overall recognition rate were 100%, 100%, 85.71%, 82.61%, and 92.08% respectively. For the PCA-SVM model established with the refractive index spectrum, the recognition rates for normal, germinated, moldy, worm-eaten wheat, and the overall recognition rate were 100%, 80.95%, 86.96%, and 91.98%, respectively. However, the corresponding recognition rates based on fusion models reached up to 90%. The experiments indicate that, for wheat samples of different quality, the multi-source information fusion, established through a combination of the absorption spectrum and the refractive index spectrum, improves the recognition rate of wheat. More specifically, the recognition rate using the RBF-SVM feature fusion is the highest, suggesting it is the optimal method of multi-source information fusion. In order to further demonstrate the validity of the proposed method, the obtained results were examined in terms of the measurement time. Each sample was measured five times to generate an average spectrum, where the scan time was no more than five s. Thus, the prediction time for each sample is no more than six s (including the total scan time and the calculation time of one second) on a standard PC with four GB of RAM and a Pentium CPU. The classification recognition results for each sample can be completed within six s, indicating the feasibility of the fast information fusion methods.

### 3.5. Discussion

**Figure 4:**
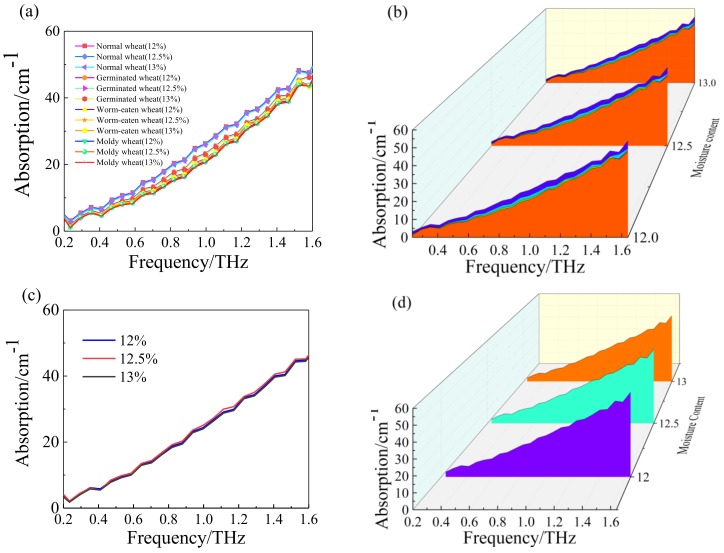
THz Absorption spectra of varying moisture content levels of different wheat grain (**a**) and waterfall plot (**b**) THz Absorption spectra of varying moisture content levels of worm-eaten wheat (**c**) and waterfall plot (**d**).

Chua et al. [38] uses the THz-TDS to measure absorption spectra of wheat grain with 8%, 12%, and 18% moisture content, it also analyzes the relationship between the moisture content and THz transmission signals. It is well known in THz frequency region that liquid water presents a strong absorption. The absorption spectra of wheat grain of varying moisture content levels (dry, 12%, 12.5%, and 13%) were calculated using THz-TDS, which associated with both free water molecules and hydration water within the grain. In order to isolate the effect of the moisture content, the spectra were subtracted from the dry wheat grain. The absorption spectra were shown in the Figure 4. It demonstrates that the samples have no obvious absorption peaks in the frequency range, and also shows that the moisture content was about 12.5%, and has little influence on the THz radiation. The differences in THz absorption for different varieties of wheat are due to the variations in the composition of different wheat samples. Guo et al. [39] reports a method of evaluation of wheat seeds by using THz imaging mainly based on the reflection mode. However, few studies report multi-source information fusion methods on the multiple optical indexes of sample for the improvement in model performance. The applicability of THz-TDS combined with multi-source information fusion technology to determine the quality of wheat grain is present in this study. Better prediction results obtained were comparable with previous reports in the region of 0.2–1.6 THz. In particular, it is of great concern for agricultural products and food safety, particularly packaged goods. THz wave has the ability to penetrate a wide range of materials (such as paper, plastic, and cloth). Given this characteristic, we will continue to explore the quality of packaged wheat and examined the effect of packaging materials by using THz-TDS in the future.

In addition, the absorption spectrum was used in the literature [23], although a satisfactory result can be obtained by the constructed model. The fusion optical parameter (the absorption spectrum and the refractive index spectrum) provides rich information based on the molecular structure. In our work, these preliminary results indicated that THz-TDS combined with DS evidence theory analysis was suitable for the determination of the storage induced quality defects with better detection accuracy. However, the influencing factors of the spectra (such as plant areas, etc.), will need to be considered in further studies. Further research (including the development of a cost-effective THz spectrometer with high SNR and assay) is required to improve the robustness of the prediction model for real application in the agricultural products/food. Furthermore, the determination of lower levels of the defective samples would be of great value, as well as the further quantitative analysis of wheat grains of different germination times and stages of mold growth, and studies in which the defective samples are blended with the control wheat at ratios from 1:9 through to 9:1 in order to build to more valuable models to discriminate varieties.

## 4. Conclusions

In this paper, samples of normal, germinated, moldy, and worm-eaten wheat were investigated. Through combination of the absorption and the refractive index spectrum, SVM and DS evidence theory with different kernel functions were used to establish a classification fusion model for the multiple optical indexes of wheat. Our modeling results indicate that the recognition rates of fusion models for wheat can reach 90%, and the recognition rate of the RBF-SVM fusion model can reach 97.5%. To verify the validity of the fusion model, the model was compared with the PCA-SVM single-spectrum analysis regression model as reported in the literature. The result shows that the fusion model has a better prediction effect with respect to the recognition rate for wheat samples of different quality. The results indicate that the fusion model improves the detection accuracy for several indexes of wheat quality defects. Among the investigated fusion models, the RBF-SVM fusion model has the highest detection accuracy; therefore, it is the currently best multi-source information fusion method. The multi-source information fusion model of the THz spectrum can solve the recognition problem for wheat quality, which is of great significance for grain quality detection.

## Figures and Tables

**Table 1:** System parameters of the Z3 THz-TDS (time domain spectroscopy).

Performance Index	Parameter Values
Pump Source	Femtosecond fiber laser
Pumping capacity	<10 nJ
Spectral range	0.1–3.5 THz
Frequency domain resolution	<5 GHz
Longest time delay	1.3 ns
Dynamic range	>70 dB (peak value)
THz radiation source	LT-GaAs photoconductive antenna
THz detector	ZnTe electro-optic crystal

**Table 2:** Modeling results of the radial basis function support vector machine (RBF-SVM) wheat classification fusion model.

Sample Type			Determine Types				False Judgment Number	Recognition Rate of Each Type (%)	Overall Recognition Rate (%)
			Normal	Germinated	Moldy	Worm-Eaten			
Training set	Normal	38	38	0	0	0	0	100	100
	Germinated	46	0	46	0	0	0	100	
	Moldy	39	0	0	39	0	0	100	
	Worm-eaten	37	0	0	0	37	0	100	
Test set	Normal	22	22	0	0	0	0	100	97.5
	Germinated	14	0	14	0	0	0	100	
	Moldy	21	0	0	20	1	1	95.24	
	Worm-eaten	23	1	0	0	22	1	96.65	

**Table 3:** Modeling results of Linear SVM wheat classification fusion model.

Sample Type			Determine Types				False Judgment Number	Recognition Rate of Each Type (%)	Overall Recognition Rate (%)
			Normal	Germinated	Moldy	Worm-Eaten			
Training set	Normal	38	38	0	0	0	0	100	100
	Germinated	46	0	46	0	0	0	100	
	Moldy	39	0	0	39	0	0	100	
	Worm-eaten	37	0	0	0	37	0	100	
Test set	Normal	22	22	0	0	0	0	100	93.75
	Germinated	14	0	13	0	1	1	92.86	
	Moldy	21	1	0	19	1	2	90.48	
	Worm-eaten	23	1	0	1	21	2	91.3	

**Table 4:** Modeling results of Poly SVM wheat classification fusion model.

Sample Type			Determine Types				False Judgment Number	Recognition Rate of Each Type (%)	Overall Recognition Rate (%)
			Normal	Germinated	Moldy	Worm-Eaten			
Training set	Normal	38	38	0	0	0	0	100	100
	Germinated	46	0	46	0	0	0	100	
	Moldy	39	0	0	39	0	0	100	
	Worm-eaten	37	0	0	0	37	0	100	
Test set	Normal	22	22	0	0	0	0	100	90
	Germinated	14	0	12	0	1	2	92.86	
	Moldy	21	1	0	18	2	3	85.71	
	Worm-eaten	23	1	0	2	20	3	86.96	

**Table 5:** The result of the decision fusion model using the DS wheat classification model.

Sample Type			Determine Types				False Judgment Number	Recognition Rate of Each Type (%)	Overall Recognition Rate (%)
			Normal	Germinated	Moldy	Worm-Eaten			
Training set	Normal	38	38	0	0	0	0	100	100
	Germinated	46	0	46	0	0	0	100	
	Moldy	39	0	0	39	0	0	100	
	Worm-eaten	37	0	0	0	37	0	100	
Test set	Normal	22	22	0	0	0	0	100	96.25
	Germinated	14	0	14	0	0	0	100	
	Moldy	21	0	0	20	1	1	95.24	
	Worm-eaten	23	0	1	1	21	2	91.3	
